# Correlation between Magnetic and Dielectric Response of CoFe_2_O_4_:Li^1+^/Zn^2+^ Nanopowders Having Improved Structural and Morphological Properties

**DOI:** 10.3390/molecules28062824

**Published:** 2023-03-21

**Authors:** Mahwish Afzia, Rafaqat Ali Khan, Bushra Ismail, Magdi E. A. Zaki, Talal M. Althagafi, Abdulaziz A. Alanazi, Afaq Ullah Khan

**Affiliations:** 1 Applied and Analytical Chemistry Laboratory, Department of Chemistry, COMSATS University Islamabad, Abbottabad Campus, Abbottabad 22060, Pakistan; 2 Department of Chemistry, Faculty of Science, Imam Mohammad Ibn Saud Islamic University, Riyadh 13318, Saudi Arabia; 3 Department of Physics, College of Science, Taif University, Taif 21944, Saudi Arabia; 4 Department of Chemistry, College of Science and Humanities in Al-Kharj, Prince Sattam bin Abdulaziz University, Al-Kharj 11942, Saudi Arabia; 5 School of Chemistry and Chemical Engineering, Jiangsu University, 301 Xuefu Road, Zhenjiang 212013, China

**Keywords:** Li^1+^/Zn^2+^ co-doped cobalt ferrite, chemical co-precipitation method, magnetic properties, dielectric properties, AC conductivity

## Abstract

The vast applicability of spinel cobalt ferrite due to its unique characteristics implies the need for further exploration of its properties. In this regard, structural modification at the O-site of spinel with Li^1+^/Zn^2+^ was studied in detail for exploration of the correlation between structural, magnetic, and dielectric properties of the doped derivatives. The CTAB-assisted coprecipitation method was adopted for the synthesis of the desired compositions owing to its cost effectiveness and size controlling ability. Redistribution of cations at T- and O-sites resulted in the expansion of the crystal lattice, but no distortion of the cubic structure was observed, which further supports the flexible crystal structure of spinel for accommodating larger Li^1+^/Zn^2+^ cations. Moreover, an XPS analysis confirmed the co-existence of the most stable oxidation states of Zn^2+^, Li^1+^, Co^2+^, and Fe^3+^ ions with unstable Co^3+^ and Fe^2+^ ions as well, which induces the probability of hopping mechanisms to a certain extent and is a well-established behavior of cobalt ferrite nanoparticles. The experimental results showed that Li^1+^/Zn^2+^ co-doped samples exhibit the best magnetic properties at dopant concentration *x* = 0.3. However, increasing the dopant content causes disturbance at both sites, resulting in decreasing magnetic parameters. It is quite evident from the results that dielectric parameters are closely associated with each other. Therefore, dopant content at *x* = 0.1 is considered the threshold value exhibiting the highest dielectric parameters, whereas any further increase would result in decreasing the dielectric parameters. The reduced dielectric properties and enhanced magnetic properties make the investigated samples a potential candidate for magnetic recording devices.

## 1. Introduction

Spinel ferrites have attracted prodigious attention in electronic engineering, such as microwave devices and emerging transformer cores, due to their low coercivity and enhanced magnetization [1]. Presently, eco-friendly inverse spinel ferrites have emerged as a potential candidate in various fields such as energy storage devices [2], drug delivery [3], resonance imaging [4], medical diagnostics [5], high-density storage devices [6], catalysis [7], microwaves, and data storage devices [8] because of their excellent magnetic, structural, optical, and dielectric properties, as well as many other properties over time.

Interestingly, cobalt ferrite has fascinated the research community due to its high coercivity (5400 Oe), high saturation magnetization (80 emu/g) at room temperature, high magnetocrystalline anisotropy, and high thermal and chemical stability [9]. Because of the aforementioned properties, a large group of researchers have conducted investigations to improve the electrical and magnetic properties of spinel ferrites [10]. Spinel structure generally allows the inclusion of dopants, which reside on the tetrahedral (A) and octahedral (B) sites of a crystal lattice and govern the significant modification of physical properties such as electrical and magnetic properties [11]. Sharifianjazi et al. demonstrated the magnetic behavior of cobalt ferrite nanoparticles and concluded that the physical properties and performance could be improved by including proper dopants [12]. Saadia et al. fabricated Li-Cu-doped cobalt ferrite nanoparticles via a hydrothermal route to investigate the electrical and magnetic properties. It was found that the dopant content causes a dip in saturation magnetization and coercivity [13].

Cobalt ferrite has fascinated the research community due to its high coercivity (5400 Oe), high saturation magnetization (80 emu/g) at room temperature, high magnetocrystalline anisotropy, and high thermal and chemical stability [9]. On account of the aforementioned properties, a large group of researchers have carried out investigations to improve the electrical and magnetic properties of spinel ferrites [10]. Spinel structure generally allows the inclusion of dopants, which reside on the tetrahedral (A) and octahedral (B) sites of the crystal lattice, which governs the significant modification of physical properties such as electrical and magnetic properties [11]. Sharifianjazi et al. demonstrated the magnetic behavior of cobalt ferrite nanoparticles and concluded that the physical properties as well as performance can be improved by the inclusion of proper dopants [12]. Saadia et al. fabricated Li-Cu-doped cobalt ferrite nanoparticles via the hydrothermal route to investigate the electrical and magnetic properties. It was found that the dopant content causes a dip in saturation magnetization and coercivity [13].

In another study, Saadia et al. co-doped cobalt ferrite with Li-Cd via cetyl trimethyl ammonium bromide (CTAB) assisted hydrothermal method but with relatively low dielectric and magnetic properties. Based on enhanced coercivity, the author suggested the use of synthesized samples in loudspeakers and motors [14]. Mmelesi et al. prepared zinc-doped cobalt ferrite through coprecipitation and found that the synthesized nanoparticles have great potential towards photocatalytic pharmaceutical degradation and antimicrobial applications [15].

During the last few years, researchers have shown interest in the fabrication of Li-doped cobalt ferrite with proven improvements in electrical and magnetic properties. Owing to the low cost of lithium ferrite, it has been considered a well-known multi-functional material with enhanced coercivity, a high Curie temperature, moderate saturation magnetization, high resistivity, and lower sensitivity to stress, offering several applications in microwave and memory core devices [16,17,18]. Kadam et al. have reported that Li-doped cobalt ferrite exhibited increased lattice parameters and coercivity [18]. The previous literature confirms lithium ferrites’ electrically low conductive nature, resulting in low dielectric losses when the electric field is applied [19,20,21,22]. The properties of nanomaterials are greatly affected by the synthetic route adopted.

A number of fabrication methods for the synthesis of spinal ferrites, such as hydrothermal and solvothermal techniques [23], co-precipitation [24], sol-gel [25,26], polyol [27], microemulsion [28], electrochemical manipulation [23], and thermal decomposition [29], have been reported. However, the co-precipitation method is cost-effective, accurate, efficient, and does not require labour [25]. Therefore, our research work used the co-precipitation method for synthesizing Li^1+^/Zn^2+^ co-doped cobalt ferrites. CTAB—a surface directing agent and stabilizer—is used for reducing the surface tension properties among nanoparticles, providing control over particle size and desired crystallinity [30]. It is also used to inhibit agglomeration, the major constraint of magnetic nanoparticles. However, the role of CTAB still needs to be studied extensively, and it is a lucrative area, in the opinion of the authors, to explore [31].

As saturation magnetization (Ms), low coercivity (Hc), a low dielectric constant, and low dielectric losses are vital parameters for developing materials to be used in high-frequency devices and transformer cores [9], for the present research work we have selected both Li^1+^ and Zn^2+^ having octahedral and tetrahedral site preferences in spinel ferrites. It is assumed that Li^1+^ will mainly obstruct the hopping mechanism of electrons in spinel ferrite among iron ions (Fe^3+^ and Fe^2+^). At the same time, Zn^2+^ will play a significant role in enhancing magnetocrystalline anisotropy as well as saturation magnetization. Hence this study aims to improve the magnetic and dielectric characteristics by Li^1+^/Zn^2+^ co-doping on cobalt ferrite nanoparticles with a proposed chemical formula of CoFe_2−2x_Li_x_Zn_x_O_4_ (*x* = 0.1–0.5).

## 2. Results and Discussions

### 2.1. X-ray Diffraction (XRD)

**Figure 1:**
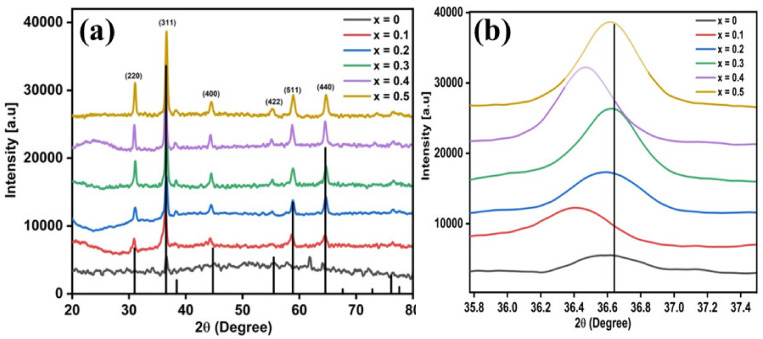
(**a**) XRD patterns of pure cobalt ferrite (*x* = 0) and doped CoFe_2−2x_Li_x_Zn_x_O_4_ (*x* = 0.1–0.5) nano powder; (**b**) Zoom of the peak corresponding to the plane (311) of doped CoLi_x_Zn_x_Fe_2−2x_O_4_ (*x* = 0–0.5) nano powder.

A spinel structure of substituted cobalt ferrites CoFe_2−2x_Li_x_Zn_x_O_4_ (*x* = 0.0, 0.1, 0.2, 0.3, 0.4, 0.5) fabricated by a low-cost chemical co-precipitation route has been investigated by X-ray diffraction. The perceived peaks are well matched with the JCPDS cards (00-022-1086) and could be allocated to the miller indices of (220), (311), (400), (422), (511), and (440), which endorse the successful synthesis of a single cubic spinel structure with no secondary phases as displayed in Figure 1a. The lattice parameter (a) of each characteristic peak of prepared samples can be calculated from the XRD data by using the following formula [32]:

(1) $$a=d{\left[\left(h^{2\;}+k^{2}+l^{2}\right)\right]}^{1/2}$$

In Equation (1), ‘hkl’ are the Miller indices, and ‘d’ is the inter planar distance of crystal planes. As shown in Table 1, the calculated lattice parameter increases from 8.3 to 8.5 Å with the substituted ions, which perfectly follow Vegard’s law [33]. According to this law, the dissimilarity of the ionic radii of substituted and replaced ions is mainly responsible for the variation in the lattice parameter ‘*a*’. Thus, the replacement of smaller Fe^3+^ (0.64 Å) [34] with large ionic radii ions such as Zn^2+^ (0.82 Å) [34] and Li^1+^ (0.74 Å) [32] causes an increase of the lattice parameter ‘*a*’. From Table 1, it is evident that cell volume ‘V_cell_’ increases gradually with increased dopant (Li^1+^/Zn^2+^) content, resulting in an increase in the lattice parameter. By considering the line broadening plane (311), the Debye-Scherer classical formula is implemented for the calculation of the average crystallite sizes of all studied samples [32]:

(2) Dhkl=0.9λβcosθ 

where the wavelength of X-rays and full width at half maximum of the corresponding peak are denoted by ‘*λ*’, and ‘*β*’ respectively. As shown in Table 1, the average crystallite size of all the prepared samples was found in the range of 23 to 16 nm due to the larger ionic radius of Zn^2+^ and Li^1+^ at both octahedral and tetrahedral lattice sites; hence, lattice strain is expected to produce dopants and disorder in the spinel lattice structure of ferrites that obstruct the grain growth and consequently the size of the nanoparticles decreases. The explanation of the smaller crystallite size of the doped sample could be attributed to the fact that Zn^2+^ and Li^1+^ are incorporated in the inverse crystal lattice of cobalt ferrite and therefore establish bonds with oxygen atoms of cobalt ferrite. This seems to indicate that the rate of nucleation of Fe^3+^-O^2^ lowers due to the inclusion of Zn^2+^ and Li^1+^ doping, thus indicating that dopants affect the crystallite size of the synthesized sample; a similar trend in crystallite size is presented in already published literature [35].

From the XRD pattern, it is found that at higher diffraction angles, peak positions are slightly shifted towards lower 2θ values with increasing dopant (Zn^2+^ and Li^1+^) content owing to the expansion of lattice parameters [35], indicating the successful incorporation of dopants into spinel ferrite. A closer look at Figure 1b shows shifting of peak position in XRD patterns, which is an indication of altered unit cell dimensions.

The following formula has been applied for the calculation of the X-ray density of all the synthesized ferrite samples [32]:

(3) $$\rho =\;\frac{8M}{N_{A}V_{cell}}$$

where, ‘*M*’, ‘*N_A_*’, and ‘*V_cell_*’ represent molar mass, Avogadro’s number, and lattice constant, respectively. The calculated X-ray density decreases with the dopant concentration (Li^1+^/Zn^2+^), as shown in Table 1, which is expected to be due to the gradual decrease in molar mass of the synthesized sample [34].

### 2.2. Morphological Analysis

**Figure 2:**
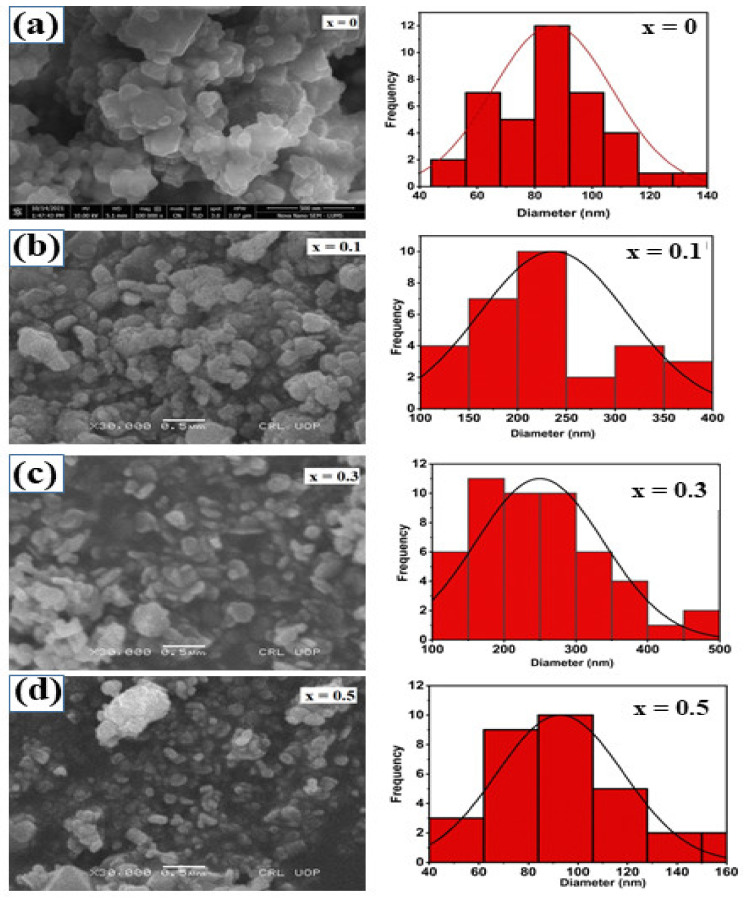
(**a**–**d**) SEM images and average particle size distribution of pure cobalt ferrite (**a**) *x* = 0 and Li^1+^/Zn^2+^ doped cobalt ferrite (**b**) 0.1, (**c**) 0.3, (**d**) 0.5.

The morphology of selected materials with desired electric and magnetic characteristics was investigated by scanning electron microscopy (SEM) for a specific application. Figure 2a–d represents the SEM micrographs of un-doped and doped cobalt ferrite with nominal compositions of CoFe_2_O_4_, CoLi_0.1_Zn_0.1_Fe_1.8_O_4_, CoLi_0.3_Zn_0.3_Fe_1.4_O_4_, and CoLi_0.5_Zn_0.5_Fe_1_O_4_, respectively. The obtained micrographs have shown that there is no uniformity in the size and shape of undoped ferrite particles. In addition, dopant has little effect on morphology, and boundaries seem unclear in undoped samples. From the SEM analysis, it is observed that micrograins of Li^1+^/Zn^2+^ co-doped nanoferrites are intermingled with each other. Furthermore, the phenomenon of coagulation seems to decrease with the inclusion of dopants. It can be seen clearly from Figure 2c that the shape of the grains is like that of plates having a rougher surface and a larger thickness, which would be due to the accumulation of metal oxide grains on the surface of CoLi_0.3_Zn_0.3_Fe_1.4_O_4,_ which can be proved from XPS analysis [36]. The doped samples (*x* = 0.5) of cobalt ferrite (CoLi_0.5_Zn_0.5_Fe_1_O_4_) are smaller, while un-doped cobalt ferrite shows agglomeration. This statement is proved with the help of XRD, which presents the smallest average crystallite size for the highest dopant concentration (CoLi_0.5_Zn_0.5_Fe_1_O_4_), i.e., 16 nm, as can be seen in SEM. It indicates that Li^1+^/Zn^2+^ content are growth inhibitors. The grain size follows the trend of crystallite size, but the magnitude is somewhat different as crystallite constitutes grains. The previous literature revealed that the magnetic properties of nanoparticles dominate effectively when the size is in the critical nano range (10–20 nm) and become a potential candidate in various applications [37]. The average particle size distribution was calculated using image J software, which shows that average particle size increases with dopant content.

### 2.3. XPS Analysis

**Figure 3:**
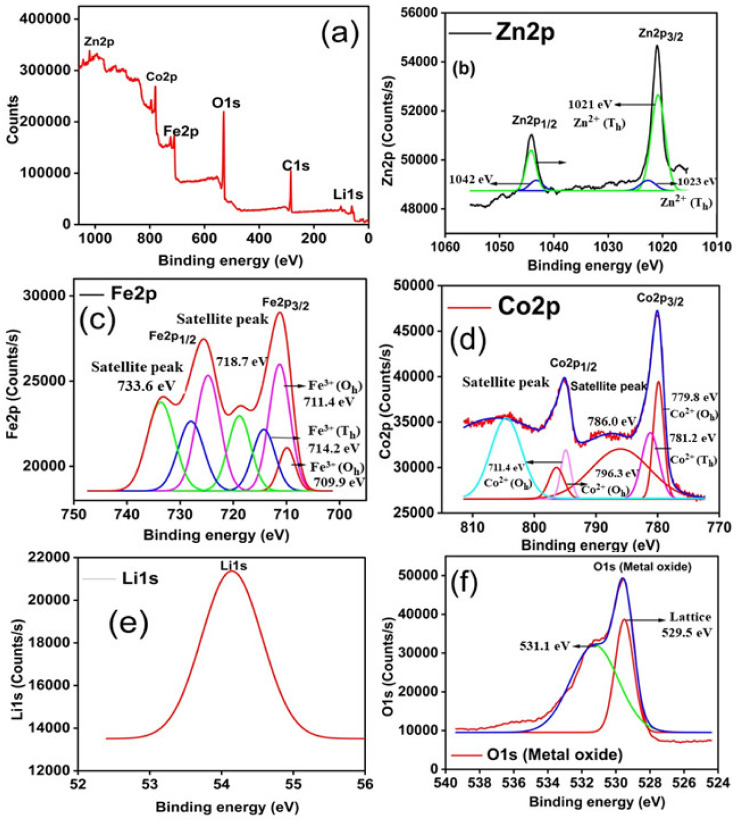
High-resolution XPS spectra (**a**) survey for CoLi_0.3_Zn_0.3_Fe_1.4_O_4_ (**b**) Zn2p (**c**) Fe2p (**d**) Co2p, (**e**) Li 1s, and (**f**) O1s of CoLi_0.3_Zn_0.3_Fe_1.4_O_4_ synthesized at optimum temperature.

The oxidation state of each element in a sample with Li^1+^/Zn^2+^ doping content *x* = 0.3 can be determined by XPS analysis. Figure 3a shows the spectrogram of CoLi_0.3_Zn_0.3_Fe_1.4_O_4_ ferrite. A closer look at the XPS spectrum Figure 3b–f of individual element shows that 2p energy level of individual element is split into two levels that is 2p_3/2_ and 2p_1/2,_ due to spin-orbit interaction [38]. Another use of XPS is the detection of two sub-lattice positions (the T_h_ and O_h_ sites) in a ferrite sub-lattice. It has been found that cations are distributed between two different positions in the crystal lattice simultaneously. The identification of different elements and their oxidation states is determined through binding energy.

Figure 3b presents two main peaks, Zn 2p_3/2_ and 2p_1/2,_ with binding energies of 1020 eV and 1043 eV, respectively, confirming that the most stable oxidation state of Zn is +2, as reported in the literature [38,39,40]. The binding energy of Zn 2p_3/2_ indicates a peak around 1021 eV and 1023 eV in tetrahedral (T_h_) and octahedral (O_h_) sites, respectively [41]. It is quite evident from Figure 3c that deconvolution of Fe (2p) envelops resulted into five characteristic peaks, located at binding energies of 710.2, 712.0, 717.7, 725.3, and 733.6 eV. Accordingly, Fe 2p_3/2_ and Fe 2p_1/2_ spin orbit components centered at 710.2 and 725.3 are due to contributions for Fe^3+^ in octahedral and tetrahedral sites, respectively. Moreover, it is revealed that the satellite peak at about 717.7 eV represents the occurrence of Fe^2+^ [42] at the octahedral site. The two weak peaks at 717 and 733.6 eV, referred to as satellite peaks, are formed as a result of electronic transitions occurring between Fe-ions during ferrite synthesis [38,43].

Figure 3d illustrates five distinct peaks of cobalt at 779.3, 781.2, 786.0, 795.4, and 804 eV. The fitting peaks at 779.3 and 781.2 are indexed to Co^2+^ in octahedral and tetrahedral sites, respectively [44,45]. Furthermore, two obvious peaks referred to as satellite peaks, found at 786.0 and 804 eV, are accounted for in the shakeup excitation of high spin [45]. Meanwhile, the weak satellite peak at 795.4 eV corresponds to the existence of low spin Co^3+^ at the octahedral site as compared to high spin Co^2+^, which may be due to the existence of an unpaired valence electron found in the orbital of Co^3+^ according to literature [42,46,47]. Additionally, a low energy band found in Figure 3e at 54 eV is related to Li 1s at the octahedral site within the nanoferrite, while the absence of a peak at 55 eV indicated that most of the Li ions are present at the octahedral site rather than the tetrahedral site [48]. Figure 3f depicts the core level spectrum of O 1s in ferrite. The main distinct peak at 529.9 eV has been assigned to lattice O^2-^, which could be due to metal oxide [47,49,50]. A second peak observed at higher binding energy, i.e., 531.5 eV, is attributed to the hydroxyl group adsorbed on the surface of ferrite, as reported in the literature [41,49].

### 2.4. Magnetic Properties

**Figure 4:**
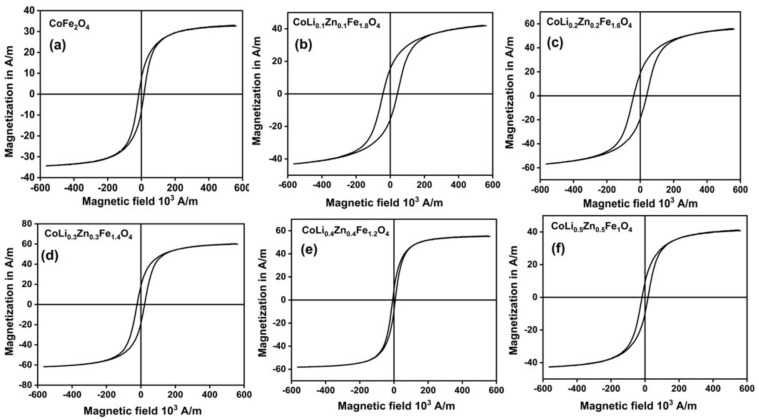
(**a**–**f**). Hysteresis loop for pure cobalt ferrite (x = 0) and doped CoLi_x_Zn_x_Fe_2−2x_ O_4_ (x = 0.1–0.5) at room temperature.

Figure 4a–f depicts magnetic hysteresis loop of the Li^1+^/Zn^2+^ doped samples obtained from vibrating sample magnetometer at the applied field of 6kA/m at room temperature. Magnetic parameters like saturation magnetization (M_s_), coercivity (H_c_) and remanent magnetization (M_r_) were calculated from magnetic loop and are listed in Table 2.

In our research work, the saturation magnetization of un-doped cobalt ferrite is about 34 A/m which is somewhat more than that of recently reported work 31.46 A/m [51]. For Li^1+^/Zn^2+^ co-doped cobalt ferrite system, the saturation magnetization initially increases at the molar ratio from 0.0 to 0.3 and is found to decrease at a ratio of 0.4–0.5 as depicted in Figure 4. Neel two sub-lattice model is used for determining cation distribution among two sites (octahedral and tetrahedral). Since Zn^2+^ and Li^1+^ are diamagnetic and paramagnetic in nature respectively with 0 *μ*_B_ (magnetic moment), so the contribution to magnetization mainly originates from magnetic ions with magnetic moments Fe^3+^ (5 *μ*_B_) and Co^2+^ (3 *μ*_B_). In crystal sublattice, octahedral site is occupied by Fe^3+^ [52], Li^1+^ [53], Co^2+^ [15,54] and tetrahedral site by Zn^2+^ [52,53,55] and Fe^3+^ [52] as already discussed in XPS analysis. Additionally, Li^1+^ and Zn^2+^ will influence the ions occupancy among two sites such that the net magnetic moment will be affected, ultimately altering the magnetization values. Initially non-magnetic Zn^2+^ substitute Fe^3+^ at tetrahedral site, where some of Fe^2+^ ions change into Fe^3+^ in this way charge neutrality is balanced [52,53]. Consequently, dilution of Fe^3+^ at tetrahedral site weakens the antiferromagnetic coupling between octahedral and tetrahedral sites, thus net magnetic moments would be increased. Therefore, at higher dopant content, net magnetic moment and super-exchange interaction are responsible for drop of saturation magnetization. 

Similarly, net magnetic moment per molecule increases at octahedral site thus, magnetization increases which is the reason for enhancement of saturation magnetization from 34 A/m to 61 A/m as ‘x’ content increased from 0.0 to 0.3. Another reason for the enhancement of saturation magnetization at content x = 0.3 is the existence of Co^3+^ (4 *μ*_B_) ions at octahedral site as per XPS result, which decreases with dopant concentration due to shifting of some Fe^3+^ and Co^2+^ towards tetrahedral site when excess Zn^2+^ moves toward octahedral site [56]. The value of saturation magnetization started to drop at x = 0.4–0.5, excess amount of Zn^2+^ ions tend to displace Fe^3+^ ions at octahedral site which lead to weakening of A-B interactions, owing to drop of net magnetic moment [57,58]. Thus, our experimental results are well explained on basis of above theory. In addition, it is noteworthy that the nanosize effect and Curie temperature may also contribute to the drop of saturation magnetization, especially for samples with high dopant content [56,59]. It is noticed from the previous reports that high concentration of Li^1+^/Zn^2+^ doping decreases Curie temperature due to the cationic redistribution (Fe^3+^) between octahedral and tetrahedral sites [60].

According to Liu et al., magnetic characteristics of doped iron (Fe) oxide would vary with Zn^2+^ (dopant) concentration. At lower concentration in tetrahedral site, Zn^2+^ ions may tend to displace some of Fe^3+^ ions to octahedral (O_h_) site resulting in increase of saturation magnetization. Above the certain limit of Zn^2+^ substitution, saturation magnetization starts to decrease because of movements of excess Zn ions to octahedral site and replace the Fe^2+^ at octahedral site [61]. Bindu et al., previously reported that redistribution of Zn^2+^ ions among both sites (tetrahedral and octahedral) was confirmed by Rietveld analysis for high Zn^2+^ concentration. Such phenomenon occurred due to creation of localized anti ferromagnetic interaction between the ions, which ultimately results in reduction of net magnetization samples [62,63]. 

However, as discussed in XPS analysis that some oxides are present on the surface of CoLi_0.3_Zn_0.3_Fe_1.4_O_4_ and potentially metal oxide (Co_3_O_4_) might be responsible for enhanced spin-orbit coupling which causes the rise in magnetocrystalline anisotropy. On further increasing the dopant content above x = 0.3 as shown in Table 2, decreased the anisotropy constant which is attributed to decrease of Co^2+^ ions at B-site. Moreover, continuous enhancement of coercivity with incorporation of Li^1+^/Zn^2+^ dopant regardless of arbitrary dissimilarity in crystallite size could only be explained in light of magnetocrystalline anisotropy. The anisotropy constant initially rises till x = 0.3 and then falls with Li^1+^/Zn^2+^ content, same results are observed in the previously reported work [64]. 

Thus, from the above discussion it is concluded that overall magnetic behavior of cobalt ferrite nanoparticles has been converted into hard magnetic ferrites with Li^1+^/Zn^2+^ doping at content of x = 0.3 whereby becoming a suitable candidate for implementation in various application such as in loudspeaker. For higher concentration of dopant, x = 0.4 and x = 0.5 displays minimum coercivity values 10 kA/m and 20 kA/m respectively. According to one-ion model, presence of Co^2+^ ions at octahedral site (O_h_) in cobalt ferrite is primarily responsible for strong anisotropy. By considering structural analysis, large ionic size of Li^1+^ and Zn^2+^ as compared to Fe^3+^ ions when doped in crystal produces lattice strain ultimately results in enhancement of magnetocrystalline anisotropy [65]. For instance, remanent magnetization Mr is closely associated with magnetocrystalline anisotropy. In our results, remanent magnetization seems to increases rapidly with addition of Li^1+^/Zn^2+^ dopant. 

### 2.5. Dielectric Measurements

The dielectric constant demonstrates the performance of localized electric charge carriers to recognize polarization mechanism. The relation (4) is applied for evaluation of dielectric constant of palletized samples of Li^1+^/Zn^2+^ co-doped cobalt ferrite (x = 0.0–0.5) prepared by low cost co-precipitation technique [14].

ε′ = 𝐶𝑑/𝐴є˚(4)

where, ‘C’ is the capacitance (in Farad), ‘d’ is the thickness (in meter), є˚ constant of permittivity of free space (8.8 × 10^−12^ F/m) and ‘A’ is the area of the pallet (A = πr^2^). The value of dielectric constant decreases with an increase in applied electric field frequency which follows the normal dielectric dispersion trend. At higher frequencies, the accumulation possibilities of charge carriers drop as it becomes difficult for charge carriers to follow the frequency of applied field, thus dielectric constant (ε′) falls. 

Generally, at low frequencies, polarization mechanism is used to explain the dispersion including four types of polarization such as electronic, ionic, dipolar and interfacial polarization [14,66,67]. Electronic and atomic polarization play active role at high frequency region while dipolar and interfacial polarization becomes significant at low frequency region [68]. However, it is found that at a region of lower frequencies, dispersion is mainly due to interfacial polarization. Such type of behaviour can be demonstrated by two-layer Maxwell-Wagner’s interfacial theory (constitute well conducting grain and poor conducting grain boundaries) which is generally in line with Koop’s theory for dielectrics [66,69]. Additionally, XPS studies showed the presence of iron (Fe^2+^ ↔ Fe^3+^) and cobalt (Co^2+^ ↔ Co^3+^) ions at octahedral sites which are responsible for hopping mechanism. Generally, at low frequencies, hopping is carried out between Fe^2+^ ↔ Fe^3+^ and between Co^2+^ ↔ Co^3+^ when the external field is applied. Accordingly, grain boundaries become charge carriers assembling region due to their high resistance, resulting in space charge polarization with high values of dielectric constant (ε′). At higher frequencies, the accumulation possibilities of charge carriers drop, subsequently polarization and dielectric constant (ε′) fall gradually [69]. According to Bajaj, S., et al. [70], the polarization phenomenon is similar to conduction mechanism. The presence of iron ions (Fe^2+^/Fe^3+^) manifests ferrite materials dipolar. 

In current report, un-doped sample CoFe_2_O_4_ exhibits maximum dielectric constant (ε′ = 5.24) at 1.5 GHz. At first the increase for sample x = 0.1 (ε′ = 5.89) could be attributed to the incorporation of Zn ions at tetrahedral site that pushes more Fe^3+^ towards octahedral site. Thus, hopping mechanism with oxygen vacancy and stress increases which leads to more production of Fe^2+^ ions in octahedral sites, which is in accordance with previous reports [71]. However, a decreasing trend is followed with dopants concentration and strain as well. Whereas CoFe_1_Li_0.5_Zn_0.5_O_4_ has the lowest dielectric constant value (2.83), at this point more Zn^2+^ ions migrate to octahedral site resulting in decreased Fe^3+^ ions at octahedral site. Li^1+^ ions are not considered to contribute in conduction route but may obstruct the motion of charge carriers [72]. 

**Figure 5:**
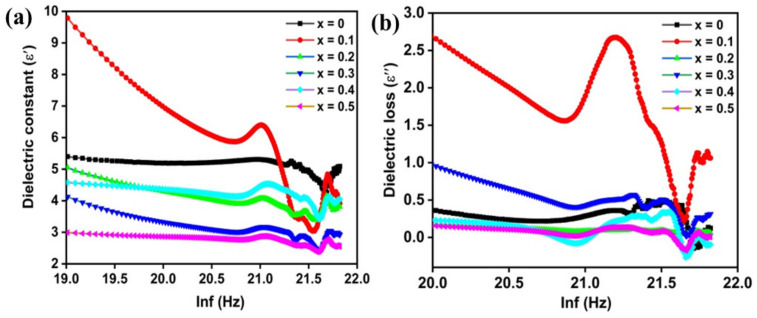
(**a**) Variation of dielectric constant (**a**) ε′ and (**b**) ε′′ with frequency for pure cobalt ferrite (x = 0) and doped CoFe_2−2x_Li_x_Zn_x_O_4_ (x = 0.1–0.5) at room temperature.

Figure 5b demonstrates the variation of dielectric loss with respect to frequency presenting a similar trend as that of dielectric constant. It can been seen in Table 2 that at 1.5 GHz frequency, sample x = 0.1 has shown highest dielectric loss. As mentioned in previous sections that as Zn^2+^ are incorporated at tetrahedral sites, it displaces few of Fe^3+^ ions towards octahedral site, which ultimately increases hopping probability at B-site (octahedral site). On the contrary, increase in dopant content (Li^1+^/Zn^2**+**^) follow decreasing trend in case of dielectric loss due to decrease of hopping possibilities at B-site. When dopant content increase, it causes the cationic redistribution among both A and B-site. In higher dopant content (x = 0.5), it might be expected that few of Zn^2+^ ions move towards B-site and displace few of Co^2+^ as well as Fe^3+^ ions towards A-site, decreasing hopping possibilities at B-site.

A close observation of Figure 3b (XPS analysis) indicates that there is some content of Zn^2+^ found at octahedral site and Co^2+^ at tetrahedral site which might increase with dopant concentration, resulting in fall of dielectric loss as well. Electronic and atomic polarization play active role at high frequency region while dipolar and interfacial polarization becomes significant at low frequency region [68,73]. Although maximum loss is achieved at lower frequency (0.5 GHz) when frequency of applied field is considerably lower than the frequency generated as a result of hopping of electron (Fe/Fe) at O-site, which follows the applied field. While at higher frequency (1.5 GHz), the case is reverse [74,75,76].

### 2.6. AC Conductivity

**Figure 6:**
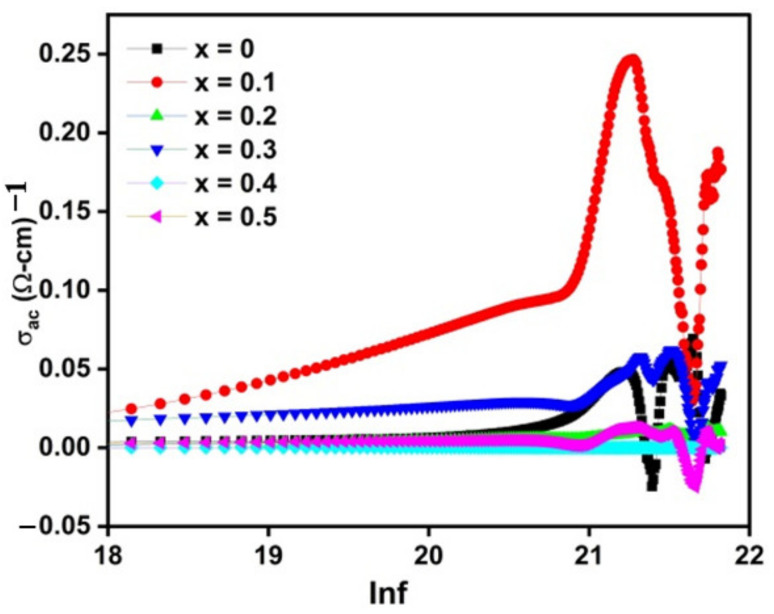
Variation in AC conductivity with frequency for pure cobalt ferrite (*x* = 0) and doped CoFe_2−2x_Li_x_Zn_x_O_4_ (*x* = 0.1–0.5) at room temperature.

Figure 6 illustrates the frequency independent behavior at low frequency region whereas there is reverse trend at high frequency region. It is proposed that at high frequencies regime, ac conductivity is increased due to hopping between Fe^2+^ ↔ Fe^3+^ and Co^2+^ ↔ Co^3+^ cations at octahedral site. As pointed out earlier that Fe^2+^ and Co^3+^ are found only at octahedral site due to which hopping mechanism is carried out only at octahedral site. Maxwell-Wagner model and Koop’s theory are used to interpret the behaviour of frequency dependent ac conductivity [71]. 

Therefore, the ferrite nanomaterial constitutes well conducting grain and poor conducting grain boundaries and thereby referred as multilayer capacitor. The grain boundary presents an active role which display the larger value of ac conductivity [9]. Considering this model, electron hopping among iron and cobalt ions is very low at low frequency region which leads to decrease of ac conductivity. On the other hand, enhanced electron hopping between Fe^2+^ ↔ Fe^3+^ and Co^2+^ ↔ Co^3+^ ions causes ac conductivity to increase at high frequency region [9,77]. Following expression (5) is taken into account for determining the relationship of ac conductivity and frequency:

(5) $$AC=A\omega^{n}$$

In expression 5, AC symbol denotes ac conductivity, ‘ω’ denotes the angular frequency of applied field, ‘A’ having conductivity’s units and factor ‘n’ calculated by the slope between natural log of ac conductivity and natural log of angular frequency [78]. The ac conductivity values are at 1.5 GHz, tabulated in Table 2 which shows that ac conductivity decreases with dopant (Li-Zn) except x = 0.1. Different electronic configuration of Zn, Co and Fe ions in crystal lattice might be responsible for variation in ac conductivity. Considering the current report, Zn^2+^ ions were incorporated in place of Fe^3+^ ions. Under the application of AC field, Zn^2+^, Co^2+^, and Fe^3+^ ions exhibit few aspects such as: (i) Zn^2+^ ions have the tendency to lose an electron which is very rare. (ii) Co^2+^ ions can be easily transformed into Co^3+^ ions (iii) Fe^3+^ ions showing strong capability to get converted into Fe^2+^ after gaining of an electron. Thus, this is a well-established behaviour presented by cobalt and iron ions, already reported in literature [62,79,80].

For sample x = 0.1, AC conductivity is maximum due to addition of Zn^2+^ ions at tetrahedral site, displacing few of Fe^3+^ from tetrahedral site which leads to increase in hopping at octahedral sites (B-site) among Fe^2+^ ↔ Fe^3+^ and Co^2+^ ↔ Co^3+^ ions. As discussed earlier, hopping can easily be carried out among ions at B–B site. Furthermore, addition of Li^1+^/Zn^2**+**^ at both sites disturbed the cations distribution (Fe^3+^) among A and B site, decreasing the electron hopping probability at B-site. Consequently, decreasing the Fe^3+^ ions at octahedral site and pushing them to tetrahedral site, leads to decrease in ac conductivity. The reduced dielectric properties and enhanced magnetic properties make the investigated samples potential candidate for magnetic recording devices. 

## 3. Experimental Section

### 3.1. Chemicals and Experimental Methods

The reagents and chemicals used in the present research work include Co(NO_3_)_2_⋅6H_2_O (>98.99%, Strem Chemicals, Newburyport, MA, USA), Fe(NO_3_)_3_⋅9H_2_O (>99.99%, Sigma Aldrich, St. Louis, MO, USA), LiCl (Sigma Aldrich 98.0%), Zn(NO_3_)_2_.6H_2_O (>99.99%, Sigma Aldrich), CTAB (C_19_H_42_BrN, Sigma Aldrich), and NaOH (>98%) and were purchased from Sigma-Aldrich. All the solutions were prepared in ultrapure water (Fischer Scientific, Philadelphia, PA, USA).

### 3.2. Synthesis of CoFe_2−2x_Li_x_Zn_x_O_4_

Li^1+^/Zn^2+^ doped cobalt spinel nanoferrites CoFe_2−2x_Li_x_Zn_x_O_4_ (*x* = 0.0–0.5) were prepared via the co-precipitation route. The respective metal nitrates and chlorides, in a stochiometric ratio of 1:2, were dissolved in distilled water. The mixture was heated under vigorous stirring with 100 mL surfactant (CTAB) at about 353 K for a few minutes to ensure complete homogenization. 0.1 M CTAB (100 mL) was used to prevent agglomeration and oxidation from atmospheric oxygen. Next, the pH of the solution was maintained at 12 by dropwise addition of a 3 M NaOH (base/mineralizer) solution. The whole solution was allowed to stir for 2 h at ambient temperature. The obtained product was washed with distilled water and dried at 353 K for 24 h, followed by grinding to powder form using a ceramic mortar and pestle. Finally, the synthesized powders were annealed in a muffle furnace at 1073 K to attain single-phase purity.

### 3.3. Characterization

The crystalline structure and phase purity were analyzed by X-ray diffraction patterns (XRD) using a Phillips X’Pert PRO 3040/60 that employs CuKα radiations. Surface morphology was analyzed using a tungsten filament-based scanning electron microscope (VEGA3 LM, TESCAN, and the Czech Republic). X-ray photoelectron spectroscopy (XPS) analysis was used for surface oxidation states and cationic distribution of the nanoparticles on ESCALAB-250 with a monochromatic Al-Kα X-ray (150 W). The energy for the survey is 200 eV, while 30 eV is for high-resolution scans. The vibrating sample magnetometer (VSM-100, Dexing Magnet Tech. Co., Limited, Xiamen, China) was used to calculate magnetic parameters at room temperature. Furthermore, dielectric parameters were studied through an (RF) impedance/materials analyzer (4291B, Agilent, CA, USA) ranging from 1 MHz to 3 GHz.

## 4. Conclusions

In this study, the successful fabrication of Li^1+^/Zn^2+^ doped cobalt ferrites was carried out via a co-precipitation route. The introduction of dopant content leads to increased lattice parameters, whereas the inclusion of Li^1+^/Zn^2+^ content in cobalt ferrite displays a prominent effect on both the structural, magnetic, and dielectric characteristics. The crystallite sizes lie between 23 and 16 nm. In addition, dopant has little impact on morphology and boundaries, which seem to be unclear as observed in doped samples. The XPS spectra confirmed the existence of the most stable (Co^2+^, Zn^2+^, and Fe^3+^) and less stable (Co^3+^ and Fe^2+^) cations of cobalt, zinc, and iron ions in the crystal structure of doped cobalt ferrite. The Li^1+^/Zn^2+^ content of 0.3 are our threshold value, and the decreasing trend is observed in the magnetic parameters at this value. This study also indicated that un-doped sample CoFe_2_O_4_ exhibits a maximum dielectric constant (ε’ = 5.24) at 1.5 GHz; at first, it increases for sample *x* = 0.1 (ε’ = 5.89), then follows the decreasing trend with dopants content. The calculated dielectric values lie in a range of 1.5 GHz to 3 GHz, indicating that doped materials are applicable in microwave devices. More importantly, the as-synthesized nanoparticles could be considered potential candidates for application in high-frequency devices, as the nanoparticles exhibit low dielectric loss. Thus, the results demonstrate that the compositional variation of AC conductivity, dielectric loss, and dielectric constant indicates a decreasing trend with the inclusion of Li^1+^/Zn^2+^ content.

## Figures and Tables

**Table 1:** Structural parameters calculated for pure cobalt ferrite (*x* = 0) and doped CoFe_2−2x_Li_x_Zn_x_O_4_ (*x* = 0.1–0.5) nano powder.

Parameters	Li-Zn Content					
	0.0	0.1	0.2	0.3	0.4	0.5
Crystallite size (nm) (XRD)	23	22	20	19	17	16
Lattice constant (Å)	8.335	8.337	8.339	8.410	8.430	8.500
Cell volume (Å^3^)	579.0	579.4	579.8	594.0	599.0	607.0
X-ray density (g/cm^3^)	5.20	5.10	5.02	4.82	4.69	4.47

**Table 2:** Electrical (at frequency 1.5 GHz) and magnetic parameters of pure cobalt ferrite (*x* = 0) and doped. CoFe_2−2x_Li_x_Zn_x_ O_4_ (*x* = 0.1–0.5) nano powder.

Parameters	Li-Zn Content					
	0.0	0.1	0.2	0.3	0.4	0.5
Dielectric constant (ε′)	5.24	5.89	3.96	3.13	4.50	2.83
Dielectric loss (ɛ″)	0.34	2.52	0.09	0.49	0.15	0.12
Ac conductivity, σ_AC_ (Ω-cm)^−1^	0.042	0.21	0.007	0.041	0.012	0.010
Saturation Magnetization, *M_s_* (A/m)	34.0	42.4	56.21	61.0	56.45	41.7
Remnant Magnetization, *M_r_* (A/m)	8.15	15.3	18.1	18.5	10.34	11.1
Coercivity, *H_c_* (10^3^A/m)	30	42	43	44	10	20

## Data Availability

Not applicable.

## Sample Availability

Samples of the compounds are not available from the authors.
